# Evaluating the effect of rutin on contrast-induced nephropathy in rats

**DOI:** 10.22038/ajp.2025.25936

**Published:** 2025

**Authors:** Faezeh Esparham, Fatemeh Rajabian, Mahboobeh Ghasemzadeh Rahbardar, Bibi Marjan Razavi, Abolfazl Khajavi Rad, Sakineh Amoueian, Hossein Hosseinzadeh

**Affiliations:** 1 *Department of Pharmacodynamics and Toxicology, School of Pharmacy, Mashhad University of Medical Sciences, Mashhad, Iran*; 2 *Pharmaceutical Research Center, Pharmaceutical Technology Institute, Mashhad University of Medical Sciences, Mashhad, Iran*; 3 *Targeted Drug Delivery Research Center, Pharmaceutical Technology Institute, Mashhad University of Medical Sciences, Mashhad, Iran*; 4 *Department of Physiology, Faculty of Medicine, Mashhad University of Medical Sciences, Mashhad, Iran*; 5 *Neurogenic Inflammation Research Center, Mashhad University of Medical Sciences, Mashhad, Iran*; 6 *Pathology Department, Emam Reza Hospital, Mashhad University of Medical Sciences, Mashhad, Iran*

**Keywords:** Diatrizoate, Rutin, Nephropathy, Oxidative stress, Kidney

## Abstract

**Objective:**

Contrast-induced nephropathy is a common cause of acute kidney injury, and oxidative stress plays an important role in its development. The flavonoid rutin is of interest for its potential antioxidant properties. This study aimed to assess the protective effects of rutin against contrast-induced renal toxicity in rats.

**Materials and Methods:**

Eight groups of male Wistar rats (n=6 in each group) were designed: (1) Sham, (2) Premedication-control (N(ω)-nitro-L-arginine methyl ester (L-NAME, 10 mg/kg, i.p.)+indomethacin (10 mg/kg, i.p.)), (3) Contrast medium (L-NAME+indomethacin+diatrizoate (12.5 ml/kg, i.p)), (4-6) Rutin (25, 50, and 100 mg/kg, p.o., for 7 days)+L-NAME+indomethacin+ diatrizoate, (7) N-acetylcysteine (NAC, 125 mg/kg, i.p.), L-NAME+indomethacin+diatrizoate, and (8) Rutin-alone (100 mg/kg). All study groups except for the sham and rutin-alone were subjected to 48 hr of water deprivation. On day 8, blood and kidney samples were isolated to evaluate oxidative stress, biochemical and histopathological changes.

**Results:**

The levels of serum blood urea nitrogen (BUN), creatinine, and malondialdehyde (MDA) were raised by diatrizoate, while glutathione (GSH) levels in renal tissue were reduced. Rutin (25, 50, and 100 mg/kg) improved biochemical parameters and oxidative stress. Diatrizoate also resulted in interstitial edema, medullary congestion, proteinaceous casts, and severe tubular necrosis in kidney tissue. Rutin (100 mg/kg) reduced tubular necrosis and interstitial edema but had no significant effect on the formation of medullary congestion and proteinaceous casts in renal tissue.

**Conclusion:**

Oxidative stress triggered by contrast-induced nephropathy is caused by a rise in MDA and a decline in GSH amounts. Rutin protects kidney tissue against contrast-induced damage through its antioxidant effect.

## Introduction

Acute kidney injury caused by contrast media administered intravenously or intra-arterially during diagnostic or therapeutic medical interventions, is known as contrast-induced acute renal injury or contrast-induced nephropathy (Rajabian et al. 2023; Topaloğlu et al. 2019). Another negative effect of contrast media has been described as chemical hypersensitivity (Iordache et al. 2019). Up to 15% of patients might require short-term dialysis although contrast-induced nephropathy is reversible. Contrast-induced nephropathy has a mortality rate ranging from 3.8 to 64%. Patients with diabetes mellitus and chronic kidney disease with reduced kidney function are at significant risk of developing contrast-induced nephropathy. Advanced age, congestive heart failure, hypertension, hyperuricemia, and volume depletion are additional factors that can increase the prevalence of contrast-induced nephropathy by up to 25% (Kusirisin et al. 2020). The underlying pathophysiology mechanisms of contrast-induced nephropathy remain poorly understood. At present, several processes have been proposed, counting direct and indirect effects besides the formation of reactive oxygen species (ROS.). High osmolality contrast media can have direct deleterious effects on nephrons comprising renal tubular endothelial and epithelial cells, which can result in mitochondrial malfunction, cellular apoptosis or necrosis, and interstitial inflammation. By altering renal hemodynamics, contrast agents can indirectly cause medullary hypoxia by causing intrarenal vasoconstriction (Mehran et al. 2019). Regarding the formation of ROS, contrast media can either increase the amount of ROS produced or decrease the activity of antioxidant enzymes, which increases oxidative stress and impairs renal function. Moreover, medullary hypoxia increases the production of ROS, which results in mitochondrial dysfunction and oxidative stress. Overall, it is clear that oxidative stress and mitochondrial dysfunction are key factors in the pathogenesis of contrast-induced nephropathy (Heyman et al. 2010). Hence, methods that amend mitochondrial dysfunction while also reducing oxidative stress are promising targets for preventing contrast-induced nephropathy (Kusirisin et al. 2020).

Earlier studies suggested that numerous herbs or their active ingredients such as resveratrol, black seed, saffron, curcumin, thymoquinone, garlic, pomegranate, ginger, grape, silymarin, and green tea could prevent contrast-induced nephropathy or renal failure (Boozari and Hosseinzadeh 2017; Boozari and Hosseinzadeh 2021). 

Rutin is a flavonoid of the flavonol type that is present in various typical plants, including tea, buckwheat, apple, and passion flowers. Additionally, it is a crucial nutritive component of plant-based drinks and food. It is also known as quercetin-3-O-rutinoside and vitamin P. Furthermore, the primary glycoside and 3-O-rhamnoglucoside form of quercetin, the most prevalent flavonol in fruits and veggies, is rutin (Hosseinzadeh and Nassiri-Asl 2014). Antioxidant, anticarcinogenic, cytoprotective, vasoprotective, cardioprotective, and neuroprotective effects are just a few of the pharmacological properties of rutin (Janbaz et al. 2002; Javed et al. 2012; La Casa et al. 2000; Schwedhelm et al. 2003). It binds to iron ion in humans and prevents it from joining with hydrogen peroxide, which would otherwise result in the formation of a highly reactive free radical that could harm cells (Motamedshariaty et al. 2014). Several studies also disclosed the renoprotective effect of rutin in animals (Dong et al. 2023; Ganesan et al. 2020; mohamed 2021).

The effects of rutin were investigated in this work using an experimental model of nephropathy based on the ameliorative effects of rutin and the underlying pathways involved in the pathophysiology of contrast-induced nephropathy. According to the results of our literature search, this is the first study in the medical literature to investigate the efficacy of rutin (25, 50, and 100 mg/kg) in inhibiting nephropathy development in contrast-induced nephropathy model in rats.

## Materials and Methods

### Chemicals

Pharmaceutical ingredient N-acetylcysteine (NAC) was prepared from Tinab Shimi, Iran. Meglumine/sodium diatrizoate (Urografin 76%) was bought from Bayer Schering Pharma, Germany. malondialdehyde bis (dimethyl acetal), tween 20%, thiobarbituric acid (TBA), potassium chloride (KCl), and phosphoric acid were prepared from Merck, Germany. L-^N^G-Nitro arginine methyl ester (L-NAME, Cat. number: 72760), indomethacin (Cat. number: 57413), rutin, tricarboxylic acid (TCA), and 5, 50-dithiobis 2-nitrobenzoic acid (DTNB), carboxymethyl cellulose (CMC), and glutathione were purchased from Sigma-Aldrich, USA.

### Animals

In the animal room of School of Pharmacy, Mashhad University of Medical Sciences, 48 healthy male Wistar rats weighing 230-250 grams were prepared and kept in standard cages at a temperature of 23 ± 2°C, in a cycle of 12 hr of darkness and 12 hr of light. Throughout the time of storage, food and water were freely available to animals (except for the dehydration times). Unfortunately, during the study two Wistar rats in the contrast medium group died and were replaced. All animal testing was conducted in accordance with the guidelines established by the Mashhad University of Medical Sciences, School of Pharmacy ethics committee and the accepted code of ethics, IR.MUMS.PHARMACY.REC.1399.089. 

### Induction of nephropathy in rats

The induction of nephropathy by contrast media in healthy animals is challenging, and has been achieved through dehydration and the administration of nitric oxide (NO) and prostaglandin (PG) inhibitors (Kusirisin et al. 2020; Sun et al. 2014). Indomethacin and L-NAME inhibit the synthesis of PG and NO, respectively. When combined, indomethacin and L-NAME cause reversible renal vasoconstriction without evident tubular damage. 

In this study, rats were deprived of water from the 5^th^ to the 7^th^ day (for 48 hr). Then, 40 hr after the onset of water deprivation, L-NAME (10 mg/kg, intraperitoneally ( i.p.)), indomethacin (10 mg/kg, i.p.), and contrast medium (diatrizoate, 12.5 ml/kg, i.p.) were administered separately with 15 min intervals and water deprivation was continued for another 8 hr for the rats (Topaloğlu et al. 2019).

### Preparation of medication administration

Carboxymethyl cellulose (CMC) and Tween 20% were used as the suspending agents for rutin and indomethacin, respectively. Rutin was suspended in deionized water (DIW) using 0.5% CMC, while indomethacin was suspended in DIW using tween 20%. Additionally, L-NAME dissolves well in NS and the diatrizoate contrast medium is a ready-to-use product.

The maximum volume of injection for L-NAME, indomethacin, N-acetylcysteine (NAC) and gavage for rutin was 0.5 ml. Furthermore, the volume of a single dose of the diatrizoate contrast medium, according to the rats' body weight, was approximately 2.5 ml.

### Experimental groups

1- Sham group: The animals in this group were not subjected to dehydration; the purpose of designing this group was to assess the effects of solvents. Thus, instead of rutin, the animals received DIW plus 0.5% CMC via oral administration (p.o.), for 7 days. On the seventh day of the experiment, instead of indomethacin, the rats received DIW plus 30 µl of Tween 20% (i.p.), and instead of L-NAME and diatrizoate contrast medium, NS (i.p.) was administered at the same volume and frequency. 

2- Premedication-control group: Rats received DIW plus 0.5% CMC instead of rutin at equal volume for 7 days. On the fifth day after starting the study, the rats were dehydrated for 48 hr. On the seventh day, they received indomethacin (10 mg/kg, i.p.), L-NAME (10 mg/kg, i.p.), and NS instead of diatrizoate contrast medium (i.p.) (Topaloğlu et al. 2019).

3- Contrast medium group: DIW plus 0.5% CMC was administered orally for 7 days. Then, rats were dehydrated from day 5 to day 7 (48 hr). On the seventh day, they were given L-NAME (10 mg/kg, i.p.), and after 15 min intervals, indomethacin (10 mg/kg, i.p) was administered. Fifteen minutes later, a diatrizoate contrast medium (12.5 ml/kg, i.p.) was injected (Topaloğlu et al. 2019). 

4-6- Rutin (25, 50, and 100 mg/kg) treatment groups: Rutin was administered at the mentioned doses for 7 days. On the seventh day of the study, indomethacin (10 mg/kg, i.p.), L-NAME (10 mg/kg, i.p.), and diatrizoate contrast medium (12.5 ml/kg, i.p.) were injected with 15 min intervals. In this study, rutin dosages were selected based on previous research (Gelen et al. 2021; Kamalakkannan and Prince 2006; Oboh et al. 2019). The rats were exposed to dehydration for 48 hr (from the 5^th^ to the 7^th^ day).

7- NAC (125 mg/kg) group, as a positive control group: The administration of different substances was the same as in groups 4-6, with the difference that the rats in this group received NAC (125 mg/kg, i.p.) instead of rutin for 7 days. The dose of NAC was chosen based on our pilot study.

8- Rutin-alone (100 mg/kg) group: Rats were given rutin (100 mg/kg, p.o.) combined with 0.5% CMC for seven days. This group was developed to study the effects of rutin-alone. This group had unlimited access to water.

**Figure 1:**
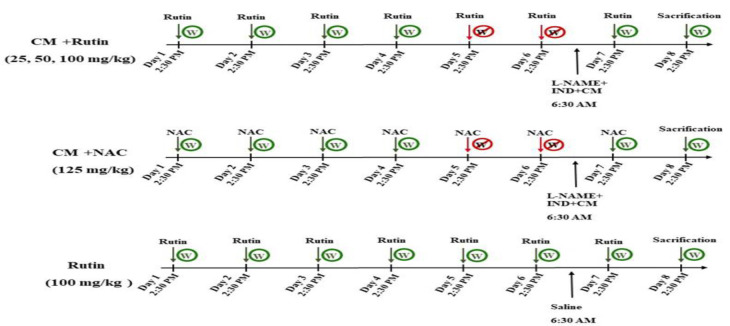
Study protocol in different experimental groups (n=6 rats in each group). CM: Diatrizoate contrast medium, CMC: Carboxymethyl cellulose, DIW: Deionised water, L-NAME: N(ω)-nitro-L-arginine methyl ester, IND: indomethacin, NAC: N-acetylcysteine

All rats in the various groups were given access to water on the seventh day of the research, 8 hr after the injection of L-NAME, indomethacin, and diatrizoate contrast medium. There was no administration on the last day of the experiment (eighth day), and the rats were sacrificed, and blood and kidney samples were collected. The right kidney of each rat was stored in liquid nitrogen and then, placed in a freezer at -80^ᵒ^C. The left kidney was fixed in 10% formaldehyde for histological analysis. It should be noted that there were six rats in each group (Figure 1).

### Renal histopathology

The sections of paraffin-embedded kidney tissue blocks were dyed with Hematoxylin and Eosin (H&E). Semiquantitative analysis of tissue sections was done blindly. Renal tissue damage was assessed for tubular necrosis, proteinous cast, medullary congestion, and interstitial edema, and scored 0-4 (0: no damage, 1: damage < 25%, 2: damage 25-50%, 3: damage 50-75%, and 4: damage > 75%) (Kongkham et al. 2013; Makhdoumi et al. 2018; Topaloğlu et al. 2019).

### Evaluating the kidney function

Blood serum samples were analyzed to assess renal function. Serum creatinine and blood urea nitrogen (BUN) levels were measured by an autoanalyzer. BUN levels were measured by the SL-urea assay kit and the Enzymatic-UV-kinetic method. Creatinine levels were also assayed using SL-creatinine assay kit and the Jaffe-kinetic method (Mindray BS-800M) (Ephraim et al. 2020; Javadi et al. 2014).

### Determining the amount of malondialdehyde (MDA)

Lipid peroxidation can be indicated by the amounts of MDA, and its elevation suggests lipid peroxidation. Accordingly, MDA reacts with TBA to produce a pink complex with a maximum absorption wavelength of 532 nm (Mihara and Uchiyama 1978).

In this study, MDA was measured manually in the laboratory. First, a 10% homogenate of the rat kidney was prepared with 1.15% cold KCl using an average of 100 mg from each tissue sample. Then, 0.5 ml of this homogenate was combined with 1 ml of 0.6% TBA solution and 3 ml of 1% phosphoric acid. For 45 min, the tubes holding this mixture were submerged in a pot of boiling water. The previously cooled mixture was mixed with 4 ml of n-butanol and vortexed for one minute to extract the colored complex. After centrifugation for 10 min at 3000 rpm, the organic supernatant was transferred to new tubes, and the absorbance was recorded for each sample at 532 nm. A standard curve was drawn for MDA concentrations between 0 and 100 nmol/ml, and malondialdehydbis (diethyl acetal) was used as the MDA standard. The MDA concentration was recorded in terms of nmol/g tissue (Rahbardar et al. 2022).

### Determining the content of reduced glutathione (GSH)

Free sulfhydryl groups react with DTNB (Ellman's Reagent) in an alkaline medium as the foundation for this test. The maximum absorbance of the colored complex produced occurs at 412 nm (Moron et al. 1979).

In this study, GSH was measured manually in the laboratory. First, each kidney tissue sample with typical weights of 100 mg was separated, and then, a 10% homogenate was prepared with phosphate buffer (pH 7.4). The samples were then combined in a 1:1 ratio with 10% TCA and centrifuged at 2500 g for 10 min. Following separation, the top phase was combined with 2 ml of phosphate buffer (pH 8). The absorbance of different samples at a wavelength of 412 nm was measured after adding 0.5 ml of 0.04% DTNB reagent. A standard curve was drawn using glutathione in the concentration range of 0-300 nmol/ml of GSH, and its amount is reported in terms of nmol/g tissue to calculate the amount of GSH (Ghasemzadeh Rahbardar et al. 2020).

### Statistical analysis

Prism 8 software was utilized for statistical computations. The pathology findings are reported as Median ± interquartile range (IQR), and the non-parametric Kruskal Wallis test and Dunn's post-test were used for data analysis. The findings of biochemical parameters and oxidative stress are presented as Mean ± SD (standard deviation) and analyzed by one-way ANOVA and the Tukey-Kramer test for *post hoc* analysis. Statistics were deemed significant at p<0.05. In this study, the Shapiro-Wilk normality test was used.

## Results

### Effect of rutin and diatrizoate contrast medium on blood levels of BUN and creatinine

**Figure 2:**
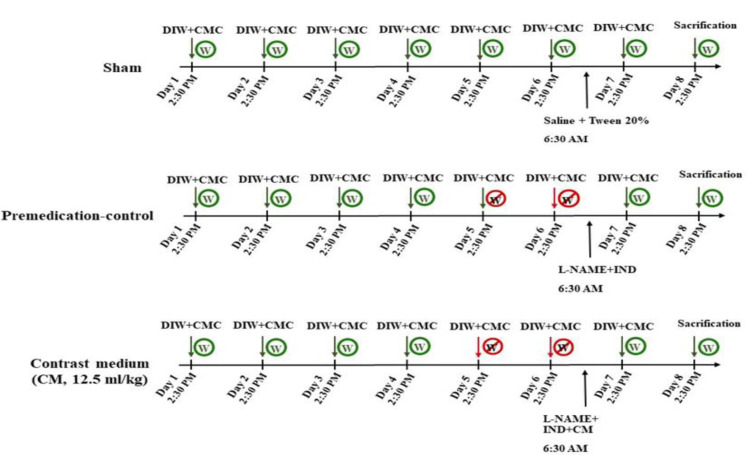
Effect of diatrizoate contrast medium and rutin on A: BUN and B: creatinine levels. Contrast medium (12.5 mg/kg), rutin (25, 50, and 100 mg/kg), and NAC (125 mg/kg) were administered to rats. Data are presented as Mean ± SD (n = 6). To assess any statistical difference, the ANOVA test and Tukey-Kramer posttest were utilized. ### p<0.001 in comparison to sham and *** p<0.001 in comparison to the contrast medium group. CM: contrast medium, NAC: N-acetyl cysteine.

The BUN and creatinine levels in the sham and control groups were identical. As shown in Figure 2A, the amount of BUN in the animals' serum increased significantly after the administration of the diatrizoate contrast medium (12.5 ml/kg) compared to the sham group (p<0.001). Simultaneous administration of rutin (25, 50, and 100 mg/kg) and diatrizoate contrast medium resulted in a significant dose-dependent decrease in BUN in the treated animals (p<0.001) versus the diatrizoate contrast medium group. Furthermore, the administration of NAC at a dose of 125 mg/kg along with diatrizoate contrast medium significantly reduced the amount of BUN caused by diatrizoate contrast medium injection (p<0.001). Rutin-alone administration at a dose of 100 mg/kg to healthy animals resulted in no significant differences compared to the sham group. The BUN amount (Mean ± SD) of sham, diatrizoate contrast medium, and rutin 100 mg/kg rutin+ diatrizoate contrast medium were 27±0.8, 150.8±11.3, and 23.5±3.2 mg/dl, respectively.

A single dose injection of diatrizoate contrast medium (12.5 ml/kg) significantly raised serum creatinine levels in comparison with the sham group (p<0.001). The rutin administration in all three doses (25, 50, and 100 mg/kg) along with the diatrizoate contrast medium, resulted in a significant reduction in creatinine in treated animals (p<0.001) versus the diatrizoate contrast medium group. On the other hand, NAC (125 mg/kg) significantly decreased serum creatinine levels compared with the diatrizoate contrast medium group (p<0.001). Administering rutin-alone (100 mg/kg) to healthy animals did not cause a significant change in the amount of creatinine compared to the sham group (Figure 2B). The creatinine amount (Mean ± SD) of sham, diatrizoate contrast medium, and rutin 100 mg/kg + diatrizoate contrast medium were 0.4±0.05, 1.4±0.4, and 0.5 mg/dl, respectively.

 Both rutin (100 mg/kg) and NAC (125 mg/kg) significantly decreased the levels of serum creatinine and BUN (p<0.001).

### Effect of rutin and diatrizoate contrast medium on MDA and GSH levels of renal tissue

**Figure 3:**
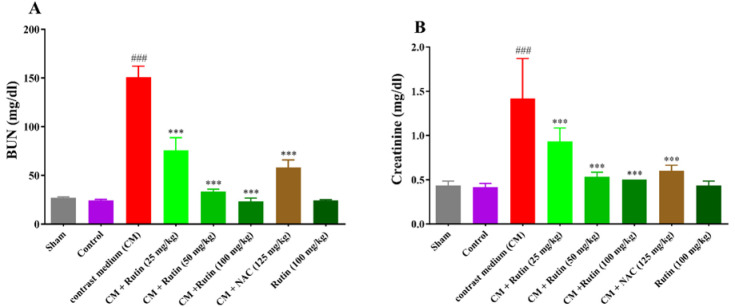
Effect of diatrizoate contrast medium and rutin on renal A: MDA and B: GSH levels. Contrast medium (12.5 mg/kg), rutin (25, 50, and 100 mg/kg), and NAC (125 mg/kg) were administered to rats. Data are presented as Mean ± SD (n = 6). To assess any statistical difference, the ANOVA test and Tukey-Kramer posttest were utilized. ### p<0.001 in comparison to sham and *** p<0.001 in comparison to the contrast medium group. CM: contrast medium, NAC: N-acetyl cysteine.

The renal tissue in the control and sham groups had the same levels of MDA. The MDA content of kidney tissue significantly increased following a single intraperitoneal injection of diatrizoate contrast medium (12.5 ml/kg) when compared to the sham group (p<0.001), while it significantly decreased following the concurrent administration of diatrizoate contrast medium and rutin (25, 50, and 100 mg/kg) compared to the diatrizoate contrast medium group (p<0.001). Administering diatrizoate contrast medium plus NAC at a dose of 125 mg/kg also resulted in a significant drop in the MDA concentration (p<0.001). Additionally, there was no noticeable change between the rutin 100 mg/kg alone group and the sham group (Figure 3A). The MDA amount (Mean ± SD) of sham, diatrizoate contrast medium, and rutin 100 mg/kg rutin+ diatrizoate contrast medium were 108.5±16.4, 185.5±14.7, and 127.6±10.9 nmol/g tissue, respectively.

When compared to the sham group, the single dose injection of diatrizoate contrast medium (12.5 ml/kg) significantly decreased the GSH level of kidney tissue (p<0.001). Compared to the diatrizoate contrast medium group, the renal GSH levels increased significantly by administrating the diatrizoate contrast medium plus rutin (25, 50, and 100 mg/kg) (p<0.001) (Figure 3B). In comparison with the diatrizoate contrast medium group, administration of NAC at a dose of 125 mg/kg along with diatrizoate contrast medium significantly increased the renal GSH concentration (p<0.001). Furthermore, there was no apparent change between the rutin 100 mg/kg alone group and the sham group. The levels of GSH in the control group significantly decreased compared to the sham group (p<0.001). The GSH amount (Mean ± SD) of sham, diatrizoate contrast medium, and rutin 100 mg/kg rutin+diatrizoate contrast medium were 301.1 ± 37.9, 243.1 ± 23.8, and 618.8 ± 11.4, nmol/g tissue respectively.

Both rutin (100 mg/kg) and NAC (125 mg/kg) significantly decreased MDA amount and increased the levels of GSH in kidney tissue (p<0.001).

### Effect of rutin and diatrizoate contrast medium on renal histology

**Figure 4:**
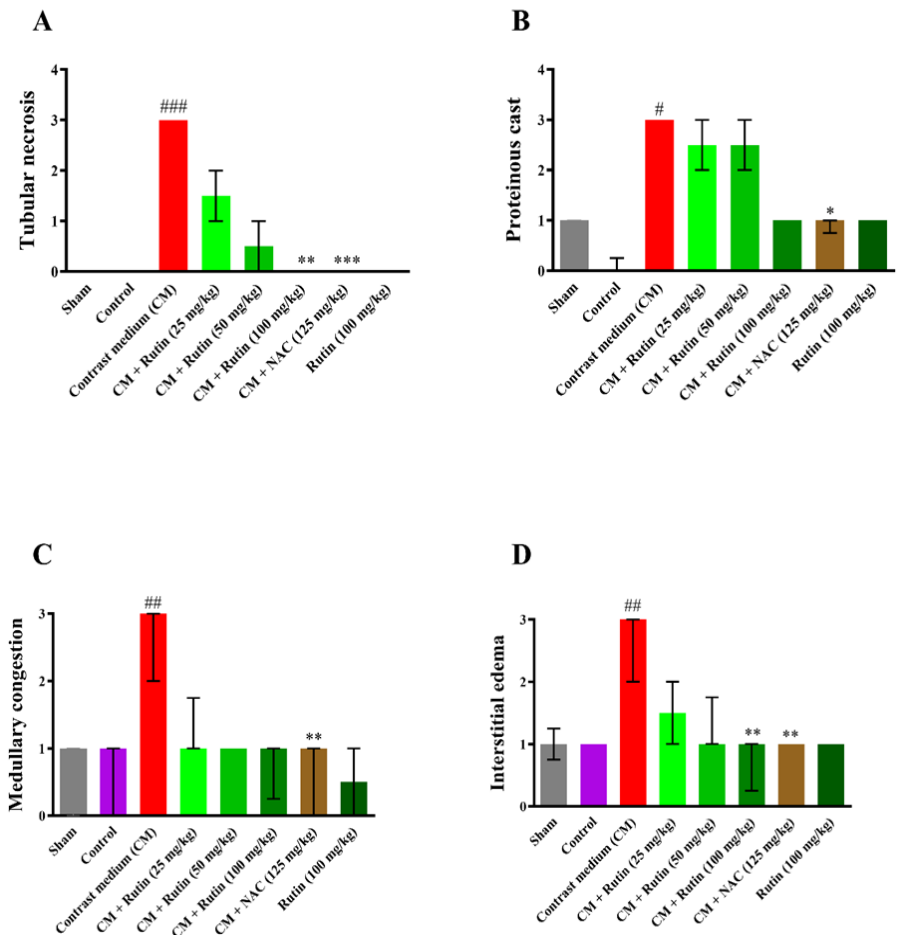
Effect of diatrizoate contrast medium and rutin on renal histology: A: tubular necrosis B: proteinaceous casts, C: medullary congestion, and D: interstitial edema. Contrast medium (12.5 mg/kg), rutin (25, 50, and 100 mg/kg), and NAC (125 mg/kg) were administered to rats. Data are presented as median ± IQR (n = 6). To assess any statistical difference, the ANOVA test and non-parametric Kruskal-Wallis test were utilized. ###p<0.001, ##p<0.01, and #p<0.05 in comparison to sham and ***p<0.001, **p<0.01, and *p<0.05 in comparison to the contrast medium group. CM: contrast medium, NAC: N-acetyl cysteine.

**Figure 5:**
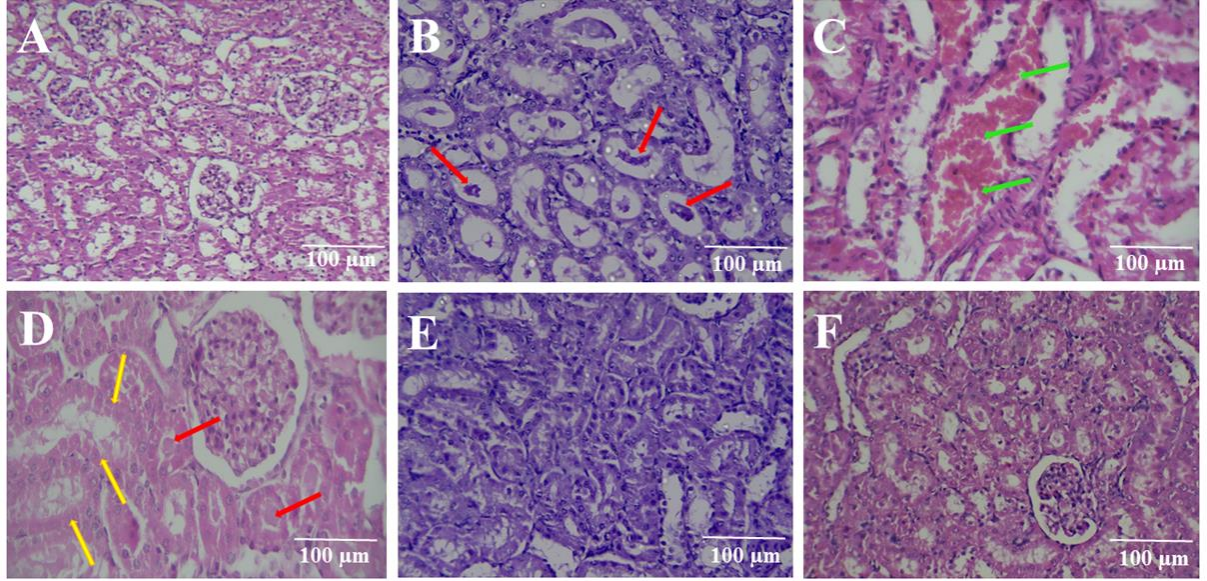
Histopathological changes of rat kidney tissue. A: sham, 10x magnification, B: CM 12.5 ml/kg (proteinaceous casts, red arrows), 40x magnification, C: CM 12.5 ml/kg (medullary congestion, green arrows), 40x magnification, D: CM 12.5 ml/kg (tubular necrosis, yellow arrows and proteinaceous casts, red arrows), 40x magnification, E: Rutin 100 mg/kg+CM 12.5 ml/kg, 40x magnification, F: NAC 125 mg/kg+CM 12.5 ml/kg, 10x magnification.

The renal histology in the control, sham, and rutin-alone (100 mg/kg) groups was identical, as shown in Figures 4 and 5.

The results demonstrated that the diatrizoate contrast medium significantly enhanced tubular necrosis when compared to the sham group (p<0.001). Rutin at a dosage of 100 mg/kg along with diatrizoate contrast medium significantly reduces tubular necrosis (p<0.01) in comparison with the diatrizoate contrast medium group, whereas doses of 25 and 50 mg/kg of rutin had no obvious impact. Additionally, when compared to the diatrizoate contrast medium group, the administration of NAC at a dose of 125 mg/kg plus diatrizoate contrast medium led to a significantly lower level of tubular necrosis (p<0.001) (Figures 4A, and 5).

In comparison with the sham group, the diatrizoate contrast medium also resulted in the formation of proteinaceous casts in the kidney tissue (p<0.05). These proteinaceous casts were present in the treatment groups that received rutin in all three doses along with a diatrizoate contrast medium (Figures 4B, and 5). The administration of NAC (125 mg/kg) besides the diatrizoate contrast medium resulted in a significantly lower level of proteinaceous casts compared to the diatrizoate contrast medium group (p<0.05). 

Compared to the sham group, the diatrizoate contrast medium induced medullary congestion in the renal tissue (p<0.01). Medullary congestion was present in the treatment groups that received rutin in all three doses along with a diatrizoate contrast medium (Figures 4C, and 5). In comparison with the diatrizoate contrast medium group, NAC (125 mg/kg) plus diatrizoate contrast medium significantly reduced medullary congestion (p<0.01).

The diatrizoate contrast medium resulted in interstitial edema in the renal tissue in comparison with the sham group (p<0.01). The interstitial edema decreased by rutin (100 mg/kg) administration along with the diatrizoate contrast medium (p<0.01), but rutin (25 and 50 mg/kg) could not decrease interstitial edema versus the diatrizoate contrast medium group. Interstitial edema significantly decreased after receiving NAC (125 mg/kg) plus diatrizoate contrast medium in comparison with the diatrizoate contrast medium group (p<0.01) (Figure 4D, and 5). 

NAC (125 mg/kg) significantly reduced all pathological changes in the kidney including tubular necrosis, proteinous cast, medullary congestion, and interstitial edema. In contrast, rutin (100 mg/kg) significantly reduced only tubular necrosis and interstitial edema. Therefore, NAC exhibited better protective effects against kidney tissue damage caused by the diatrizoate contrast medium.

## Discussion

The present study aimed to evaluate the potential renoprotective properties of rutin against contrast media-induced kidney injury in rats. The findings of this research disclosed that diatrizoate contrast medium at a dose of 12.5 ml/kg caused kidney injury in rats. The results revealed that diatrizoate contrast medium significantly augmented the levels of serum BUN, creatinine, and MDA and attenuated GSH content in rats’ kidneys. However, the concurrent administration of rutin or NAC along with a diatrizoate contrast medium reversed these changes. Furthermore, the outcomes of the pathology assessment of the rats' renal samples unveiled that the diatrizoate contrast medium injection resulted in the formation of proteinaceous casts, severe tubular necrosis, interstitial edema, and medullary congestion. Rutin at a dose of 100 mg/kg considerably reduced tubular necrosis and interstitial edema in kidney tissue but did not have a significant effect on the formation of proteinaceous casts and medullary congestion. However, diatrizoate contrast medium-induced tubular necrosis, proteinaceous casts, medullary congestion, and interstitial edema could be considerably reduced by administering NAC in addition to diatrizoate contrast medium. 

Following the administration of radiocontrast agents during diagnostic and interventional angiographic procedures, contrast-induced nephropathy (CIN) occurs. CIN is a prevalent iatrogenic hospital-acquired acute renal injury (Crimi et al. 2015; Kaliyaperumal et al. 2023) and increases hospital stay, dialysis, and mortality rates in patients (He et al. 2017). There is no effective treatment for CIN at present, thus finding a prevention strategy for CIN has great importance in its management (Li and Wang 2024). The pathophysiological causes of contrast-induced nephropathy are not fully understood (Zhou et al. 2019). Its pathogenesis has been linked to hemodynamic disturbances, medullary ischemia, oxidative stress, and direct damage to renal tubular cells (Geenen et al. 2013; Zhou et al. 2019).

According to reports, biochemical markers (BUN and Cr) are screening tests for the evaluation of kidney function (Walker et al. 1990). Our results indicated that diatrizoate contrast medium (12.5 ml/kg) along with water deprivation (for 48 hr), indomethacin (10 mg/kg), and L-NAME (10 mg/kg) increased serum creatinine and BUN levels. These results were in line with another study, that the levels of serum creatinine and BUN in rats were raised following the intravenous administration of meglumine diatrizoate 60% (6 ml/kg), indomethacin (10 mg/kg), and L-NAME (10 mg/kg) (Khaleel et al. 2019). Zhou et al. showed that the intravenous administration of ioversol (2.9 g/kg) along with indomethacin (10 mg/kg) and L-NAME (100 mg/kg) in rats enhanced the levels of serum BUN and Cr (Zhou et al. 2019). On the other hand, our data demonstrated that the administration of rutin (25, 50, and 100 mg/kg) along with the diatrizoate contrast medium significantly reduced serum BUN and creatinine levels. In another study in agreement with our obtained results, the administration of rutin (20 mg/kg, 7 days, p.o.) to guinea pigs with granuloma resulted in decreased levels of serum BUN and creatinine (mohamed 2021). Furthermore, Al-Harbi et al. reported that the oral administration of rutin at doses of 10, 20, and 40 mg/kg for 3 weeks attenuated the levels of serum BUN and creatinine and ameliorated nephrotoxicity caused by carfilzomib in rats (Al-Harbi et al. 2019). 

The enhancement of lipid peroxidation is the outcome of the nephrotoxic effects of contrast media that alter the structure of renal cells and the function of the kidney (Bolotova et al. 1993). Thus, oxidative stress is a substantial element in the development of contrast-induced nephropathy, and antioxidants might help prevent this condition (Zhou et al. 2019).

In this study, diatrizoate contrast medium (12.5 ml/kg) increased MDA levels while decreasing GSH content. Khaleel et al. showed that the administration of meglumine diatrizoate 60% (6 ml/kg, intravenous) raised the levels of MDA in rat kidney tissue (Khaleel et al. 2019). The data obtained from another study indicated that the contrast medium (sodium/meglumine amidotrizoate, 12.5 ml/kg) increased MDA levels while decreasing GSH content in rat kidney tissue (Zolfaghari Farajerdi et al. 2024). 

Rutin has antioxidant potential that acts by scavenging oxygen radicals, stabilizing cell membranes, and inhibiting DNA damage (Yang et al. 2008). In this study, rutin at doses of 25, 50, and 100 mg/kg decreased the level of MDA and increased GSH content in renal tissue. In another study, rutin (10, 20, 40 mg/kg, p.o., for 3 weeks) ameliorated carfilzomib-induced nephrotoxicity by reducing MDA levels and increasing GSH amounts in kidney tissue (Al-Harbi et al. 2019). Moreover, rutin (100 mg/kg, 7 days, p.o.) decreased the level of MDA in nephrotoxicity induced by gentamicin in rat kidney tissue (Josiah et al. 2020). Thus, the protective effects of rutin may be due to the scavenging of free radicals, decreased ROS production, and inhibition of lipid peroxidation (Iova et al. 2021). 

In the present study, pathological alterations including tubular necrosis, proteinaceous casts, medullary congestion, and interstitial edema in the kidney tissue of the diatrizoate contrast medium (12.5 ml/kg) group were observed. According to histological findings, diatrizoate (6 ml/kg, intravenous) enhanced interstitial hemorrhage, and glomerular hypercellularity (Khaleel et al. 2019). In line with our investigations, Topaloğlu et al. also observed that the administration of high osmolar contrast agent diatrizoate (along with indomethacin and L-NAME) caused proteinaceous casts, medullary congestion, and tubular necrosis in kidney tissue (Topaloğlu et al. 2019). It was reported that ioversol (2.9 g/kg) along with indomethacin (10 mg/kg) and L-NAME (100 mg/kg) could worsen pathological renal damage including tubular necrosis, medullary congestion and vacuolar degeneration in rats (Zhou et al. 2019).

The protective effect of rutin on pathological renal damage has been investigated in several studies. Rutin (10, 20, and 40 mg/kg, p.o., for 3 weeks) reduced carfilzomib-induced histological alterations such as renal tubular cell dilatation, degeneration and necrosis, and hemorrhage between medulla and cortex (Al-Harbi et al. 2019). In another study, rutin (100 mg/kg, 7 days, p.o.) ameliorated leukocyte inflammatory cell infiltration, swelling and vacuolation of the endothelium, and tubular and Bowman’s capsule dilation against gentamicin-induced nephrotoxicity in rats (Josiah et al. 2020). Similar to previous studies, we showed that rutin (100 mg/kg) lessened tubular necrosis and interstitial edema in kidney tissue. Additionally, rutin at doses of 25 and 50 mg/kg slightly reduced tissue lesions induced by diatrizoate contrast medium, however, this reduction was not significant. The reason may be that the improvement of oxidative stress and biochemical changes is insufficient to prevent pathological damage (Zolfaghari Farajerdi et al. 2024).

In this research, we used NAC at a dose of 125 mg/kg as a positive control. Our data revealed that the administration of NAC along with the diatrizoate contrast medium improved biochemical parameters (the levels of serum creatinine and BUN), oxidative stress (decreased levels of MDA and increased GSH content), and histopathological changes (interstitial edema, medullary congestion, proteinaceous casts, and tubular necrosis). Another study also claimed that the administration of NAC to rats with streptozotocin-induced diabetic nephropathy improved renal function by decreasing the levels of MDA and enhancing GSH content in kidney tissue (Mahajan et al. 2020). It has also been reported that in patients undergoing emergency percutaneous coronary intervention, rapid intravenous hydration with sodium bicarbonate and NAC is effective and safe in preventing CIN before contrast medium injection (Recio-Mayoral et al. 2007).

In the present study, all three doses of rutin and NAC showed similar effects in reducing serum creatinine and BUN concentrations, increasing GSH and reducing MDA in kidney tissue. However, NAC appeared to be much more effective than rutin in inducing histological changes.

The findings of this research show that the induction of oxidative stress via an increase in MDA and a decrease in GSH levels may result in kidney injury triggered by the contrast media. Rutin, on the other hand, lowers the tissue and biochemical alterations induced by the contrast substance through its antioxidant properties.

## Data Availability

The data that support the findings of this study are available from the corresponding author upon
